# Case Report: Nadolol–mexiletine combination therapy for refractory neonatal long QT syndrome: a case series beyond sodium channelopathies

**DOI:** 10.3389/fped.2026.1823685

**Published:** 2026-06-10

**Authors:** Jihye You, Hyun Ho Kim

**Affiliations:** 1 Department of Pediatrics, Jeonbuk National University Children’s Hospital, Jeonju, Republic of Korea; 2 Research Institute of Clinical Medicine of Jeonbuk National University, Jeonju, Republic of Korea; 3 Department of Pediatrics, Seoul St. Mary’s Hospital, College of Medicine, The Catholic University of Korea, Seoul, Republic of Korea

**Keywords:** anti-arrhythmia agents, *KCNQ1*, long QT syndrome, mexiletine, nadolol, neonatal arrhythmia, *SCN5A*, ventricular tachycardia

## Abstract

Neonatal long QT syndrome (LQTS) is associated with substantial mortality and poses significant therapeutic challenges, particularly in cases refractory to beta-blocker monotherapy. Mexiletine is established for LQT3 caused by *SCN5A* gain-of-function mutations, but its role in potassium-channel-related neonatal LQTS is limited. We present two neonates with LQTS refractory to nadolol monotherapy: one with a heterozygous pathogenic *SCN5A* c.5287G > A (p.Val1763Met) variant (LQT3), and one with a heterozygous *KCNQ1* exon 13–15 deletion (LQT1). In both cases, addition of mexiletine to nadolol was associated with QTc shortening, reduction in ectopic burden, and resolution of 2:1 atrioventricular conduction in Case 1; no adverse events were documented. We describe the clinical experience descriptively, without proposing a generalizable mechanistic claim regarding mexiletine outside LQT3. No ICD implantation was performed and no deaths occurred during follow-up. These observations support further evaluation of mexiletine as an adjunct in refractory neonatal LQTS, including non-LQT3 subtypes.

## Introduction

Long QT syndrome (LQTS) is a rare cardiac channelopathy characterized by QT interval prolongation on electrocardiogram (ECG) in the absence of structural heart disease or exogenous factors such as medications. The syndrome can manifest as episodes of syncope and, more critically, as sudden death (1–3). Congenital LQTS results from mutations in genes encoding ion channels or their modulatory proteins and occurs in approximately 1:1,500 to 1:4,350 individuals (4, 5). Neonatal LQTS is even rarer and historically carried a mortality rate approaching 50% before the age of 6 months in pre-genetic-screening era cohorts (6); contemporary cohorts in which family screening and genetic testing are systematically applied have reported severe cardiac event rates of approximately 20% (7). Post-mortem molecular autopsy studies have identified LQTS-associated variants in approximately 7.5% of cases originally classified as sudden infant death syndrome (8). Beta-blockers represent the cornerstone of treatment, and pacemaker implantation may be required in pharmacologically refractory cases (9). Nevertheless, the risk of sudden death persists even under medication, underscoring the challenge of managing neonatal LQTS. Mexiletine, a class IB antiarrhythmic agent and lidocaine congener, has emerged as a viable option for recurrent ventricular tachycardia (VT) or ventricular fibrillation (10).

Although mexiletine use in neonatal LQTS type 3 (LQT3) caused by *SCN5A* sodium channel mutations is best established (11–13), prior reports have also described mexiletine in non-LQT3 neonates, including LQT2 (7, 14). Against this background, we postulated that if nadolol proves inadequate in suppressing ectopy or controlling ventricular repolarization, mexiletine could represent a useful adjunct regardless of the underlying channelopathy subtype. Two neonates presented with severe LQTS refractory to nadolol monotherapy: one with LQT3 (heterozygous pathogenic *SCN5A* c.5287G > A, p.Val1763Met), and one with LQT1 (heterozygous *KCNQ1* exon 13–15 deletion). Both presented with markedly prolonged QTc intervals (539–542 ms) and frequent ectopic activity.

## Case presentation

### Case 1

A male infant was delivered at a gestational age of 38 + 1 weeks via emergency cesarean section because of fetal distress, with bradycardia (HR <100 bpm) noted immediately post-birth. Family history was unremarkable. Initial vital signs were: blood pressure 76/33 mmHg, heart rate 60 bpm, respiratory rate 52/min, and body temperature 36.4 °C. Physical examination revealed an irregular heartbeat without murmurs. The initial 12-lead rhythm strip on the day of birth, prior to any antiarrhythmic medication (Figure 1A), demonstrated sinus rhythm with frequent PVCs, broad T waves, and a markedly prolonged QTc of 542 ms (Bazett's correction; tangent method, lead II; HR 136 bpm). At slower sinus rates within the same recording (HR 65 bpm; QTc 503 ms), the prolonged ventricular repolarization translated into 2:1 atrioventricular conduction with blocked P waves visible within the broad T waves on the lead II rhythm strip. Same-day telemetry additionally documented dynamic, rate-dependent transitions between 1:1 and 2:1 atrioventricular conduction within the same continuous tracing (Supplementary Figure S1), supporting a functional, repolarization-dependent block mechanism rather than intrinsic atrioventricular nodal disease. Maternal anti-Ro/SSA and anti-La/SSB antibodies were negative, excluding maternal autoimmune-mediated congenital atrioventricular block.

**Figure 1:**
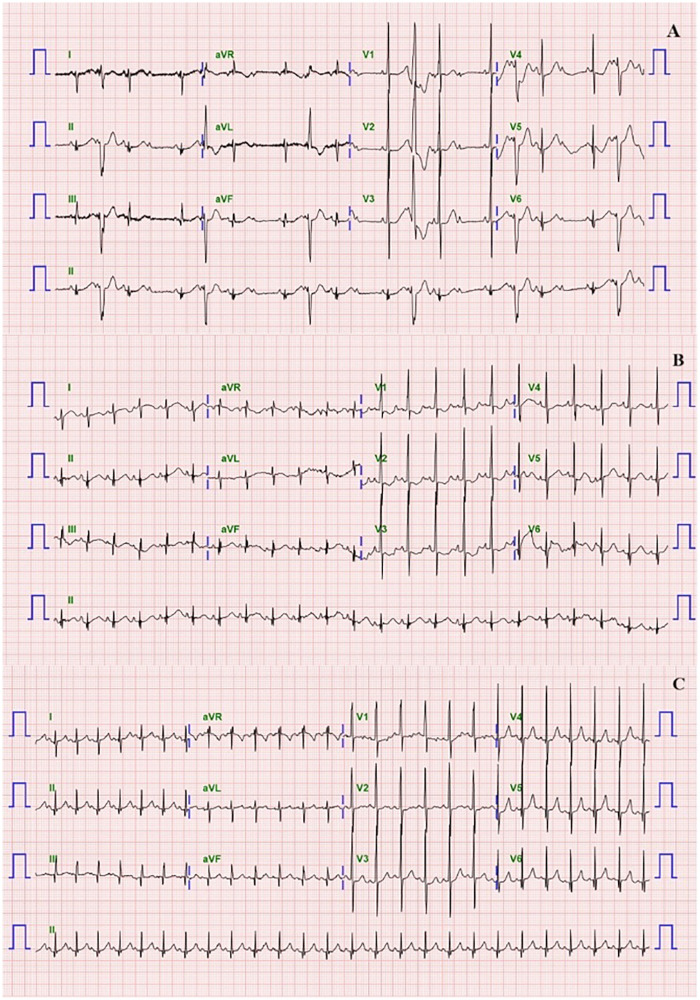
Electrocardiographic findings of case 1. **(A)** Initial 12-lead recording on the day of birth, prior to any antiarrhythmic medication: sinus rhythm with frequent PVCs, broad T waves, and a markedly prolonged QTcB of 542 ms (tangent method, lead II; HR 136 bpm). At slower sinus rates within the same recording (HR 65 bpm; QTcB 503 ms), 2:1 atrioventricular conduction emerged with blocked P waves visible within the broad T waves. **(B)** On flecainide and nadolol therapy (post-natal day 2): 1:1 atrioventricular conduction restored; HR 65 bpm; QTcB 503 ms. **(C)** One-month follow-up on nadolol and mexiletine (flecainide discontinued): normalization of QTcB to 381 ms; HR 151 bpm; sinus rhythm with 1:1 conduction. All QTc values measured by the tangent method on lead II (or V5 when lead II was not interpretable) with Bazett's correction; conducted sinus beats only.

On hospital day 2, plasma lactate rose to 5.9 mmol/L in the setting of HR ∼60 bpm, and emergency right femoral temporary pacemaker insertion was performed. Pacing maintained the set ventricular rate, but right lower extremity color change and swelling subsequently developed, with imaging confirming femoral venous thrombosis. The pacemaker sheath was removed (full removal was required because of bleeding), and the infant received heparin anticoagulation for approximately 2 days, after which the extremity findings resolved. Follow-up cranial ultrasound showed no hemorrhage. Nadolol was initiated, and flecainide was added on November 10 under a working impression that broad-spectrum sodium-channel blockade might mitigate the repolarization abnormality, prior to availability of mexiletine. On flecainide and nadolol therapy (Figure 1B; HR 65 bpm, QTcB 503 ms), 1:1 atrioventricular conduction was restored, although the QTc remained markedly prolonged. Mexiletine was introduced on November 12 at 6 mg/kg/day divided every 8 h and titrated to 12 mg/kg/day. The starting dose was selected at the lower end of the published paediatric range (5–15 mg/kg/day in three divided doses) (11, 13) given the neonatal age, the immaturity of hepatic metabolism in the first weeks of life, and the absence of an established neonatal-specific dosing reference, with subsequent upward titration to clinical and electrocardiographic effect. Doses are expressed as mexiletine hydrochloride and were administered as an extemporaneous oral preparation compounded by the hospital pharmacy, with the per-dose volume calculated individually for each patient by body weight. Flecainide was discontinued on November 19. Follow-up Holter monitoring on November 15 documented complete resolution of the 2:1 atrioventricular conduction with no further blocked beats. Mexiletine was well tolerated, with no adverse events documented. Hereditary arrhythmia panel testing (96 genes; Supplementary Table S1) identified a heterozygous *SCN5A* c.5287G > A (p.Val1763Met) variant, classified as Pathogenic per ACMG/AMP criteria with the following supporting evidence: PS3 (functional studies in patient-specific induced pluripotent stem cell cardiomyocytes), PM2 (extremely rare in gnomAD and KRGDB), PM1 (located in a critical functional region of the cardiac sodium channel), PP3 (concordant in silico predictions), and PP5 (ClinVar Variation ID 67,974, with prior reports in LQTS and cardiomyopathy patients). Maternal ECG was unremarkable; the father declined further evaluation, so segregation analysis remains incomplete. By one month of age, on continued therapy with nadolol and mexiletine, the ECG demonstrated complete normalization of the QTc to 381 ms (HR 151 bpm) with restoration of 1:1 conduction (Figure 1C). At 1.5 years of follow-up, the infant has shown normal growth and development without arrhythmic events.

### Case 2

A male infant was delivered by cesarean section at a gestational age of 38 + 3 weeks after fetal heart sonography demonstrated extra heartbeats. HR was 85/min on presentation, with an irregular heartbeat and no murmur. Growth parameters were normal, and family history of sudden death was unremarkable.

The initial 12-lead ECG on the day of birth (Figure 2A; HR 90 bpm, QTcB 539 ms) demonstrated sinus rhythm with markedly prolonged QTc, frequent PVCs, and broad-based T waves consistent with LQT1 morphology. The exceptionally wide T waves had initially given the impression of intermittent 2:1 atrioventricular block; however, on careful inspection of the rhythm strip with attention to PP intervals, conducted P waves are identifiable within and at the end of each T wave and 1:1 atrioventricular conduction was preserved throughout. A follow-up 12-lead ECG on post-natal day 3 (Figure 2B; HR 122 bpm underlying sinus rate, QTcB 542 ms) demonstrated bigeminal premature ventricular contractions with broad-based T waves and 1:1 atrioventricular conduction. Echocardiography showed a stretched patent foramen ovale with left-to-right shunt and no structural abnormality. Holter monitoring documented multifocal PVCs comprising approximately 20% of beats, predominantly in a sustained bigeminal pattern. The wide-complex beats occurred at a fixed coupling interval of approximately 360 ms with full compensatory pauses, and retrograde P waves were visible following several wide-complex beats on lead II — features collectively supporting ventricular origin and arguing against rate-dependent aberrancy. Nadolol was initiated, and flecainide was added briefly (May 31 to June 2) under a working impression that broad-spectrum sodium-channel blockade might mitigate the repolarization abnormality, prior to mexiletine availability and prior to confirmation of the LQTS diagnosis. Mexiletine was introduced on June 2 at approximately 6.0 mg/kg/day, administered as 6.5 mg per dose every 8 h, and titrated stepwise by per-dose increments (6.5, 9, 11, and 14 mg per dose three times daily), corresponding to approximately 6.0 to 12.0 mg/kg/day over approximately two weeks, with body-weight–based dose adjustments (Supplementary Table S2); the same compounded oral formulation described above was used. Serial Holter monitoring during dose titration demonstrated a stepwise reduction in PVC burden (June 6: 6%, 10,686 bigeminal cycles, single 3-beat run; June 13: 3%, 5,325 bigeminal cycles, no runs), with a partial rebound (June 19: 5%, 8,337 bigeminal cycles, no runs) prompting further escalation as body weight increased. Flecainide was discontinued on June 3, the day after mexiletine introduction. Mexiletine was well tolerated, with no adverse events documented. The hereditary arrhythmia panel (96 genes; Supplementary Table S1) identified a heterozygous *KCNQ1* (NM_000218.3) deletion encompassing exons 13–15 (chr11:2797174–2799296), confirming LQT1 with autosomal dominant inheritance. Cascade testing identified the same *KCNQ1* deletion in the mother, whose resting 12-lead ECG was unremarkable, consistent with the incomplete penetrance described in *KCNQ1*-related LQT1. By HD 31, ECG showed near-normalization of the QTc to 435 ms (HR 126 bpm; Figure 2C), and the infant was discharged on nadolol 3 mg/kg/day and mexiletine 12 mg/kg/day.

**Figure 2:**
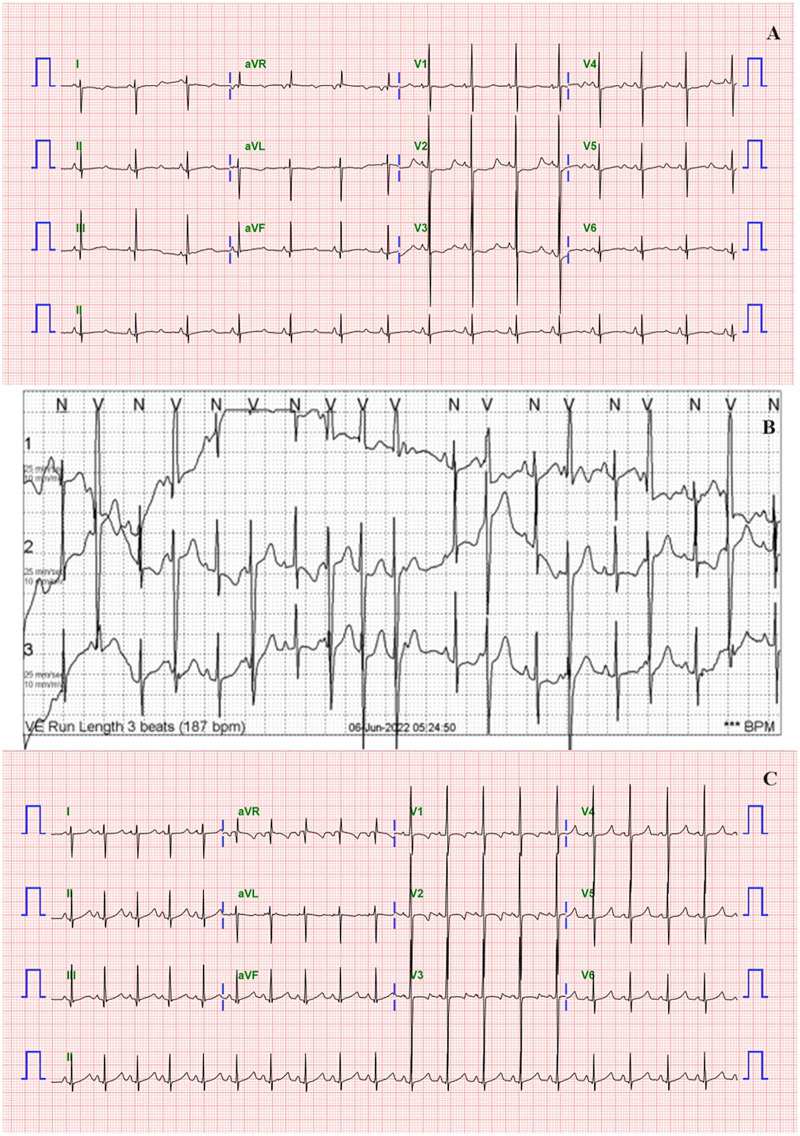
Electrocardiographic findings of case 2. **(A)** Initial 12-lead ECG on the day of birth: sinus rhythm with frequent PVCs, broad-based T waves consistent with LQT1 morphology, and 1:1 atrioventricular conduction; HR 90 bpm, QTcB 539 ms. **(B)** Post-natal day 3: bigeminal PVCs with broad-based T waves; HR 122 bpm (underlying sinus rate), QTcB 542 ms; 1:1 atrioventricular conduction preserved. **(C)** One-month follow-up on nadolol and mexiletine (flecainide discontinued): near-normalization of QTcB to 435 ms; HR 126 bpm. All QTc values measured by the tangent method on lead II (or V5 when lead II was not interpretable) with Bazett's correction; conducted sinus beats only.

One-month post-discharge follow-up demonstrated near-complete resolution of PVCs. After a stable year, mexiletine was gradually reduced and discontinued. Growth and development have remained normal.

## Discussion

To our knowledge, this is among the first reports describing the use of mexiletine in neonatal LQTS caused by potassium channel mutations (*KCNQ1*/LQT1). Although prior reports have described mexiletine primarily in LQT3 (*SCN5A* mutations) and a smaller literature exists for LQT2 (11–14), evidence for non-LQT3 subtypes in the neonatal period remains limited. This case series describes our clinical experience with the addition of mexiletine to nadolol across two distinct neonatal LQTS subtypes.

Neonatal LQTS presents with bradycardia, PVCs, and tachyarrhythmia, differing substantially from the clinical profile in older patients (6). It should be noted, however, that PVCs are not specific to LQTS and may also be observed in healthy neonates (15). A single-center Korean study reported a 10-year event-free survival of 73.2% in neonates with LQTS (16). The therapeutic challenge is intensified by the absence of well-defined neonatal treatment guidelines and by the pronounced mortality risk (6). Existing recommendations are largely extrapolated from studies in older populations (9), and the rarity of neonatal LQTS further limits the evidence base.

In our series, nadolol was used as first-line therapy (9). Non-selective beta-blockers, particularly propranolol and nadolol, attenuate catecholamine-mediated adrenergic stimulation, reducing heart rate and suppressing triggered arrhythmias (2, 9), and have demonstrated superior efficacy over atenolol and metoprolol in the management of LQTS (17). The heterogeneous response in our cohort underscores the need for a patient-specific approach accounting for both patient age and LQTS subtype.

In Cases 1 and 2, flecainide was administered briefly prior to mexiletine availability and prior to confirmation of the LQTS diagnosis. We recognise that flecainide is generally avoided in LQTS because of its IKr-blocking effect and associated pro-arrhythmic risk, and we do not recommend its use as a bridging agent in this setting. In our cases, flecainide was discontinued promptly upon introduction of mexiletine. We acknowledge that the concurrent use of multiple antiarrhythmic agents limits the ability to attribute clinical benefit to any single drug. The combined use of class IB (mexiletine) and class IC (flecainide) sodium-channel blockers also raises theoretical concerns regarding additive sodium-channel blockade and pharmacokinetic interaction, and we do not recommend routine combined use in neonates.

Mexiletine has well-established efficacy in LQT3, where it directly inhibits the gain-of-function late sodium current responsible for QT prolongation (11). Reported responses in LQT2 are variable, with a substantial proportion of non-responders (14), and although mexiletine has been shown to shorten the QT interval in LQT1 (18), evidence for an antiarrhythmic effect — i.e., reduction in arrhythmic events — in LQT1 is not yet established. Our clinical observations across two subtypes are presented descriptively; we do not propose a generalizable mechanistic claim regarding mexiletine outside LQT3 from a two-patient case series. Whether mexiletine confers benefit in non-LQT3 LQTS subtypes through effects on overall ventricular repolarization, and whether such an effect translates into a reduction in arrhythmic events, remains to be established in larger studies. We also acknowledge that, in early neonatal life, QT interval variability is substantial (19, 20), and that diagnostic reasoning in this period must integrate repeated measurements, clinical events, family history, genetic findings, and treatment response rather than relying on a single early-neonatal QTc.

The role of implantable cardioverter–defibrillator (ICD) therapy in this population warrants explicit consideration. Case 1 (LQT3, peak QTcB 542 ms, neonatal symptomatic presentation with functional 2:1 atrioventricular conduction) carries a substantial estimated arrhythmic risk and warrants individualised reconsideration of ICD indication as the patient grows and somatic constraints permit. Pharmacotherapy, including mexiletine, may shorten and stabilise the QT interval but does not substitute for ICD therapy in patients meeting established indications (9). The timing of ICD implantation in the neonatal and early-infant period is, however, constrained by patient size, lead options, and complication rates, and these factors informed our shared decision-making in the present series; ICD candidacy will be reassessed at each follow-up visit.

This series has several limitations. First, the single-center, retrospective design and small sample preclude generalizability. Second, concurrent use of nadolol, flecainide, and mexiletine in both cases limits the ability to isolate the independent contribution of mexiletine. Third, the *SCN5A* variant in Case 1 has not been confirmed by parental segregation testing because the father declined evaluation; in Case 2, the mother was a carrier of the same *KCNQ1* deletion but was phenotypically silent, consistent with incomplete penetrance. Fourth, maternal autoimmune workup was performed in Case 1 (anti-Ro/SSA and anti-La/SSB negative) but was not performed in Case 2, in the absence of clinical features suggestive of autoimmune-mediated congenital heart block.

## Conclusion

The combination of nadolol and mexiletine was associated with QTc shortening and reduction in ectopic burden in two neonates with refractory LQTS across different subtypes, including LQT3 and LQT1. No ICD implantation was performed and no mortality occurred during follow-up. These descriptive observations support further evaluation of mexiletine as an adjunct in refractory neonatal LQTS, including non-LQT3 subtypes, in larger prospective studies.

## Data Availability

The original contributions presented in the study are included in the article/Supplementary Material. Further inquiries can be directed to the corresponding author.
