# Supplementary material for: Case Report: Nadolol–mexiletine combination therapy for refractory neonatal long QT syndrome: a case series beyond sodium channelopathies
Source: Front Pediatr. 2026 Jun 10;14:1823685. doi: 10.3389/fped.2026.1823685 (PMC13290446; doi:10.3389/fped.2026.1823685)
Supplement: Supplementary file 1 [file Supplementaryfile1.docx]

**Supplementary Materials**

**Supplementary Table S1. Hereditary Arrhythmia Gene Panel (96 Genes)**

Genes included in the comprehensive hereditary arrhythmia panel performed at the participating institution. The panel is organised into **Essential Genes** (established disease-gene associations for inherited arrhythmia syndromes) and **Additional Genes** (genes with reported but less robust associations). All gene symbols follow HGNC nomenclature.

**Essential Genes (n = 31)**

| *ABCC9* | *AKAP9* | *ANK2* | *CACNA1C* | *CACNA2D1* | *CACNB2* |
| --- | --- | --- | --- | --- | --- |
| *CALM1* | *CALM2* | *CASQ2* | *CAV3* | *GPD1L* | *HCN4* |
| *KCND3* | *KCNE1* | *KCNE2* | *KCNE3* | *KCNH2* | *KCNJ2* |
| *KCNJ8* | *KCNJ5* | *KCNQ1* | *PKP2* | *RANGRF* | *RYR2* |
| *SCN1B* | *SCN3B* | *SCN4B* | *SCN5A* | *SNTA1* | *TRDN* |
| *TRPM4* |  |  |  |  |  |

**Additional Genes (n = 65)**

| *ACTN2* | *ANKRD1* | *BAG3* | *DES* | *DSC2* | *DSG2* |
| --- | --- | --- | --- | --- | --- |
| *DSP* | *EMD* | *GJA5* | *HADHA* | *JUP* | *KCNA5* |
| *LDB3* | *LMNA* | *MYH6* | *MYH7* | *NKX2-5* | *NPPA* |
| *PDLIM3* | *PLN* | *PRKAG2* | *RBM20* | *SALL4* | *SCN2B* |
| *TBX5* | *TGFB3* | *TMEM43* | *TNNI3* | *TNNT2* | *TTN* |
| *CALM3* | *CDH2* | *CPT1A* | *CTNNA3* | *DBH* | *DEPDC5* |
| *FLNC* | *GATA4* | *GATA5* | *GATA6* | *GNB5* | *GYG1* |
| *KCNA1* | *KCNE5* | *KCNK3* | *KCNQ2* | *KCNQ3* | *KCNT1* |
| *LEMD2* | *MYL4* | *NOS1AP* | *NUP155* | *PCDH19* | *PPA2* |
| *PRRT2* | *SCN10A* | *SCN1A* | *SCN8A* | *SCN9A* | *SLC25A20* |
| *SLC2A1* | *SLMAP* | *TECRL* | *TNNI3K* |  |  |

*Note:*

*All gene symbols are shown in italics in accordance with HGNC nomenclature. Variants identified in additional genes are reported only if classified as Pathogenic (PV) or Likely Pathogenic (LP). The panel uses next-generation sequencing of coding exons and adjacent intronic regions, with copy-number analysis performed when analytically supported. Deep intronic variants and large genomic rearrangements outside the validated assay scope have limited detectability with this approach.*

**Supplementary Table S2. Mexiletine Dose Escalation and Serial Holter Findings in Case 2**

Mexiletine doses are expressed as mexiletine hydrochloride. Doses were administered three times daily (TID) and titrated stepwise on the basis of clinical tolerance, ECG response, and serial Holter monitoring of premature ventricular contraction (PVC) burden. mg/kg/day values are approximate, calculated using the patient's body weight at each dose change.

| **Period** | **Mexiletine dose (per dose × TID)** | **≈ mg/kg/day** | **Holter date** | **PVC %** | **Bigeminal cycles** | **VT runs** |
| --- | --- | --- | --- | --- | --- | --- |
| Initiation (~Jun 3) | 6.5 mg TID | ~6.0 | Jun 6 | 6% | 10,686 | 1 run, 3 beats (187 bpm) |
| Jun 3 onward | 9 mg TID | ~8.0 | — | — | — | — |
| Jun 6–7 | 11 mg TID | ~10.0 | — | — | — | — |
| Jun 8–13 | 14 mg TID | ~12.0 | Jun 13 | 3% | 5,325 | 0 |
| Jun 14 | 15 mg TID | — | — | — | — | — |
| Jun 15 onward | 16 mg TID | — | Jun 19 | 5% | 8,337 | 0 |
| Jun 22 (continued) | 16 mg TID | — | Jun 22 | 6% | 14,485 | 1 run, 3 beats (139 bpm) |
| Jun 24 onward (weight-adjusted) | 17 mg TID | ~12.0 | Jun 26 | 5% | 12,176 | 0 |

*VT, ventricular tachycardia. PVC, premature ventricular contraction. TID, three times daily. Body-weight–based dose adjustments were made approximately weekly. Serial Holter findings demonstrate an initial reduction in PVC burden with mexiletine titration to 12 mg/kg/day (Jun 13, 3%; no VT runs), followed by a partial rebound (Jun 19, 5%; Jun 22, 6% with one short VT run) prompting further weight-based dose increase from 16 mg to 17 mg TID. The subsequent Holter on Jun 26 showed reduced bigeminal cycles and absence of VT runs.*

**Supplementary Figure 1. Case 1 — Pre-medication telemetry strip showing rate-dependent atrioventricular conduction**


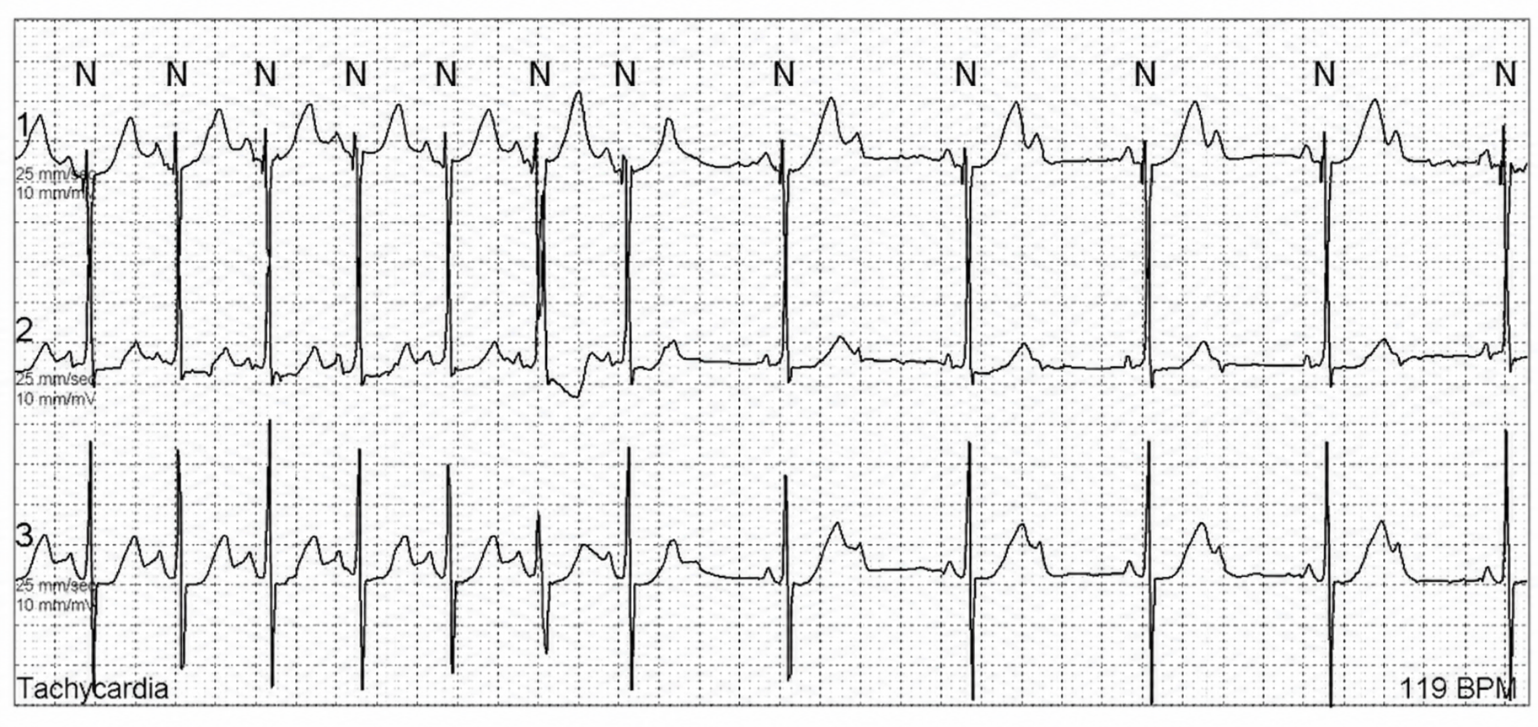


Continuous telemetry tracing recorded on the day of birth in Case 1, prior to administration of any antiarrhythmic medication (flecainide was started the following day, mexiletine two days later). At a faster sinus rate (HR 119 bpm, left portion of the strip) 1:1 atrioventricular conduction is preserved. As the sinus rate slows (HR ~65 bpm, right portion), 2:1 atrioventricular conduction emerges, with blocked P waves visible within and at the end of the broad T waves. The atrial rate is preserved while the ventricular response is halved. QTcB measured from a representative conducted beat at HR 136 bpm was 542 ms (lead II, tangent method with Bazett's correction). This rate-dependent transition between 1:1 and 2:1 conduction within the same tracing, in the absence of any antiarrhythmic medication, supports a functional, repolarization-dependent block mechanism related to extreme ventricular repolarization prolongation, rather than intrinsic atrioventricular nodal disease.
